# The Effect of the COVID-19 Pandemic and the Establishment of a Ronald McDonald House on Skin-to-Skin Times in the Neonatal Intensive Care Unit: A Retrospective Study

**DOI:** 10.3390/children12060803

**Published:** 2025-06-19

**Authors:** Stephanie Schaible, Edda Hofstätter, Wanda Lauth, Martin Wald

**Affiliations:** 1 Division of Neonatology, Department of Pediatrics and Adolescent Medicine, Paracelsus Medical University, 5020 Salzburg, Austria; stephanie.schaible@stud.pmu.at (S.S.);; 2 Team Biostatistics and Big Medical Data, IDA Lab Salzburg, Paracelsus Medical University, 5020 Salzburg, Austria; 3 Research Program Biomedical Data Science, Paracelsus Medical University, 5020 Salzburg, Austria

**Keywords:** kangaroo care, extremely premature infants, siblings, COVID-19, Ronald McDonald House

## Abstract

**Objectives**: Kangaroo care is vital for the development of premature and low-birthweight infants. However, detailed data on skin-to-skin times, especially for extremely preterm infants in NICUs, is lacking. This study quantifies skin-to-skin times for these infants at the neonatology department in Salzburg, considering factors like the COVID-19 pandemic, the opening of Ronald McDonald House, and sibling presence. **Methods**: We retrospectively analyzed data from the first eight weeks of life of 93 extremely preterm infants (<28 gestational weeks, <1500 g birth weight) treated at the Salzburg NICU from 2019 to 2023. Skin-to-skin times were recorded to the minute. **Results**: The mean value skin-to-skin time per visiting day was 241 min (±83), skin-to-skin was performed on 79.0% (±16.8) of the days of stay examined. During the pandemic, skin-to-skin care was performed on 64% of visit days, after the pandemic on 91% (*p* < 0.001). Before the Ronald McDonald House opened, the skin-to-skin time per visiting day was 215 min (±57.9), afterwards it was 273 min (±97) (*p* = 0.001). For Primipara the Kangaroo-Care time per day of visit was 257 min (±93), for Multipara 217 min (±52) (*p* = 0.043). **Conclusions**: Skin-to-skin is crucial for extremely premature infants and can be implemented for many hours a day. It is an integral part of parent-child interaction in a neonatal intensive care unit. External factors such as infrastructure, pandemic restrictions or siblings have a significant impact on skin-to-skin.

## 1. Introduction

During the last trimester of pregnancy, fundamental processes of neuronal cell growth and brain maturation take place [1]. Term-born infants born on schedule always experience a balance between stimulation and protection from overstimulation during this key developmental phase in the womb. However, if birth occurs prematurely, this balanced level of intrauterine stimulus regulation is lost [2,3]. Numerous but unregulated stimuli can have a negative impact on brain development [3]. To address these challenges of extrauterine development, special developmental programs such as the Newborn Individualized Development Care and Assessment Program (NIDCAP) have been developed [3]. These programs aim to create a more physiological environment for the developing brain. A key component of NIDCAP is direct skin-to-skin contact between parents and premature infants, which is also an essential part of “kangaroo care” [4]. Direct skin-to-skin contact helps the premature baby to adapt physically and emotionally to its new environment outside the womb [5]. The aim is to minimize the disturbance of brain development and to support its development [6]. The duration of the daily skin-to-skin time correlates with the neurodevelopmental outcome of the preterm infants [7].

The implementation of Kangaroo Care in practice is often determined by external factors [8]. For example, parents only have the opportunity to stay with their preterm infants during the night if appropriate accommodation is available for parents in or near intensive care units and they do not have to travel long distances home [9,10]. Since 2010, a parents’ refuge with four bedrooms has been available in the neonatology department at Salzburg State Hospital. However, this solution proved to be inadequate in the long term, as the rooms could not provide enough space for a week-long stay. In order to better meet the needs of parents, a Ronald McDonald Children’s Aid House with 16 residential units was opened on the grounds of Salzburg University Hospital in January 2022. The Ronald McDonald House offers temporary accommodation to parents of seriously ill children from the entire children’s hospital. In order to promote contact between parents and preterm infants, care was taken during the construction of the house to ensure that the distances to the neonatology department are short and that there is sufficient living space for the parents [11].

The time of the COVID-19 pandemic was also decisive. During this time, extensive restrictions on visiting times were introduced in many places, both inside and outside the hospital environment [12].

Despite the evidence that parental presence in neonatal intensive care units significantly improves preterm infant outcomes, there is little evidence to date that quantifies parental presence [13]. The aim of this study was therefore to describe how much time parents and extremely preterm, very low-birthweight infants spend with skin-to-skin care and what influence external factors such as the opening of a Ronald McDonald House in the immediate vicinity of the neonatal intensive care unit or the COVID-19 pandemic have on skin-to-skin times.

## 2. Materials and Methods

This retrospective data analysis was conducted at the University Hospital of Paracelsus Medical University Salzburg. Preterm infants with a gestational age of less than 28 weeks and a birth weight of less than 1500 g born between 2019 and 2023 were included. Preterm infants with morphological pathologies or abnormalities of the brain, intraventricular cerebral hemorrhage or periventricular leukomalacia of any degree, cerebral seizures and preterm infants who died during the stay were excluded.

The main outcome parameter was skin-to-skin time per visit day and the number of visit days on which skin-to-skin contact was documented. No distinction was made as to whether the skin-to-skin contact was with the mother, the father or another caregiver.

The skin-to-skin times are recorded to the minute for each visit by the ICU nursing staff using the electronic documentation program MetaVision (version 5.46.42, iMDsoft, Tel Aviv, Israel). However, electronic documentation is only accurate to the minute if the premature baby is in a neonatal intensive care unit. In Salzburg, the first skin-to-skin contact with even the smallest and most immature premature babies usually takes place immediately after birth in the delivery room or in the operating room. This usually lasts between 30 and 60 min and cannot be documented electronically. This first skin-to-skin contact can therefore not be taken into account in this study. The visits during which the extremely small premature baby is brought to the mother, e.g., if the mother also needs to be cared for in an intensive care unit, are also not recorded electronically to the minute. In addition, the documentation also changes when the premature baby is transferred to one of the family rooms directly connected to the intensive care unit. From this point onwards, the care of premature babies is largely taken over by the parents, who are present around the clock. The skin-to-skin times in the family room are therefore no longer documented to the minute. In order to avoid possible distortions in the daily skin-to-skin times, only the first eight weeks of life were taken into account, which all extremely small premature babies spend in an intensive care room.

In addition to the demoraphic data also collected on the premature infants, such as gestational week or birth weight, the presence of siblings was also recorded as a further factor influencing skin-to-skin times.

All data collected was transferred to Excel lists and stored securely within the clinic. Pseudonymization was carried out by assigning an individual four-digit identification number to each participant. Further statistical processing was carried out exclusively in this pseudonymized form.

The study was conducted as part of a diploma thesis. The responsible ethics committee Salzburg approved the study with EC No. 1166/2023 on 14 February 2024.

### Statistics

For each preterm infant, the mean duration of skin-to-skin contact per visit day and the mean number of visit days on which skin-to-skin contact occurred were calculated for each week of life individually and across all eight weeks of life studied. The mean value and standard deviation for metric variables and absolute and relative frequencies for ordinal and binary variables were used for the descriptive analysis and the description of the characteristics. The differences between the subgroups (years/COVID-19, Ronald McDonald House, siblings) were analyzed using the Mann-Whitney U test for two groups and the Kruskal-Wallis test for more than two groups. The two-sided significance level α = 0.05 is assumed for all tests. In the case of more than two groups and a significant result, the Dunn test was used as a post hoc test. The calculations were performed using the statistical software R (version 4.3.2).

## 3. Results

In total, the skin-to-skin times of 93 preterm infants were analyzed. Of these, 10 preterm infants were born in 2019, 20 preterm infants in 2020, 21 preterm infants in 2021, 24 preterm infants in 2022 and 18 preterm infants in 2023. 59 mothers of these preterm infants were primipara, 30 multipara, the information was missing for four preterm infants. The mean birth weight was 753.9 g (±207.04), the mean gestational age was 25.60 weeks (±1.48) and the mean length of stay in the intensive care unit was 66.94 days (±35.10). The overall mean value over the entire observation period shows that skin-to-skin care took place on 79% of the days (±17.8). The mean skin-to-skin time per visiting day was 242 min (±83).

**Figure 1:**
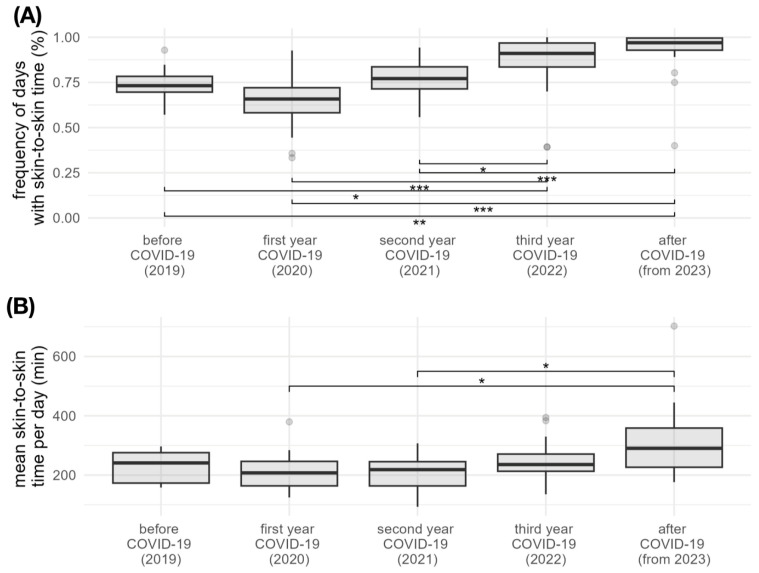
Box-whisker plots of the frequency of skin-to-skin care days (**A**) and the mean skin-to-skin time per visit day (**B**) for all years analyzed. The boxplots show the minimum, first quartile, median, third quartile and maximum. (*: *p* < 0.05, **: *p* < 0.01, ***: *p* < 0.001).

The following results were obtained for the individual years: The relative number of days with skin-to-skin time before COVID-19 (2019) was 74.6% (±9.69). In the first year of the pandemic (2020), the proportion was 64.1% (±15.07). In the second year of the pandemic (2021) 76.5% (±10.25), in the third year of the pandemic (2022) 85.82% (±16.63) and after the pandemic (2023) 91.6% (±14.62). The mean length of skin-to-skin times per visit day in 2019 was 231 min (±56). In 2020 it was 211 min (±64), in 2021 it was 213 (±55), in 2022 it was 248 min (±62) and in 2023 it was 307 min (±124). The frequency of days with skin-to-skin Care, the mean skin-to-skin time per visiting day and overall *p*-values are listed in Table 1 for all analyzed years and shown graphically in Figure 1.

Post hoc testing revealed significant differences in the frequency of days with skin-to-skin care between before COVID-19 and the third year of COVID-19 (*p* < 0.05), before COVID-19 and after COVID-19 (*p* < 0.01), the first year of COVID-19 and the third year of COVID-19 (*p* < 0.001), the first year of COVID-19 and after COVID-19 (*p* < 0.001), the second year of COVID-19 and the third year of COVID-19 (*p* < 0.05) as well as the second year of COVID-19 and after COVID-19 (*p* < 0.001). Regarding the mean skin-to-skin time per day, significant differences were found between the first year of COVID-19 and after COVID-19 (*p* < 0.05) as well as between the second year of COVID-19 and after COVID-19 (*p* < 0.05).

**Figure 2:**
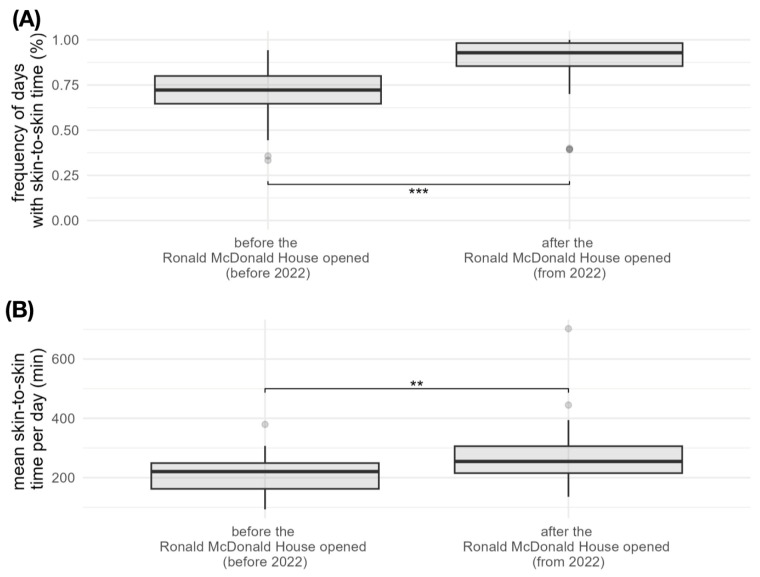
Box-whisker plot of the frequency of days with skin-to-skin care (**A**) and the mean skin-to-skin time per visiting day (**B**) before and after the opening of the Ronald McDonald House. The boxplots show the minimum, first quartile, median, third quartile and maximum. (**: *p* < 0.01, ***: *p* < 0.001).

Before the Ronald McDonald House opened (before 2022), the mean frequency of visiting days with skin-to-skin care was 71.2% (±13.40). After the opening, the mean frequency was 88.2% (±15.88) (*p* < 0.001). Before the opening of the Ronald McDonald House (before 2022), the mean duration of skin-to-skin time per visiting day was 215 min (±57.9). After the opening of the Ronald McDonald House (from 2022), the mean skin-to-skin time per visiting day was 273 min (±97) (*p* = 0.001). Mean frequency and skin-to-skin times are shown graphically in Figure 2.

**Figure 3:**
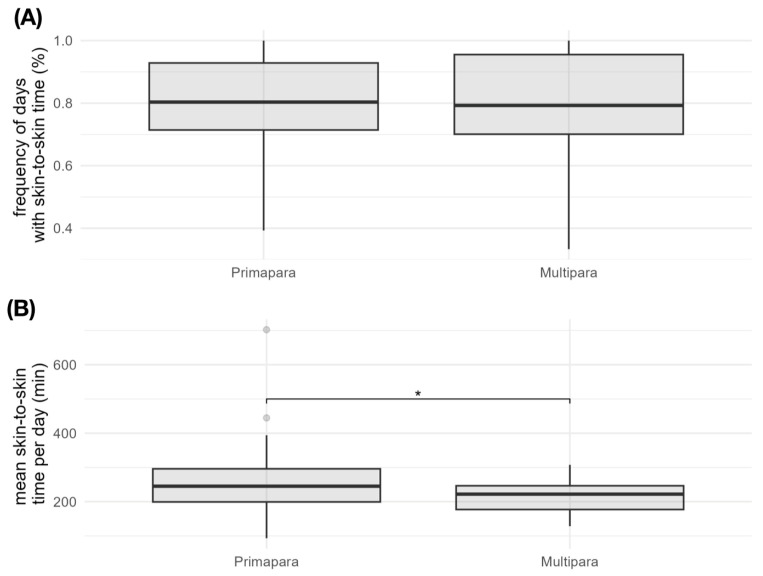
Box-whisker plot of the frequency of days with skin-to-skin time (**A**) and the mean skin-to-skin time per visit day (**B**) for Primipara and Multipara. The boxplots show the minimum, first quartile, median, third quartile and maximum. (*: *p* < 0.05).

Primipara do skin-to-skin care on 80% (±15.8), multipara on 78% (±18.3) (*p* = 0.751) of the days during the observation period. For primipara, the mean skin-to-skin time per visit day was 257 min (±93), for multipara the mean skin-to-skin time per visit day was 217 min (±52) (*p* = 0.043). Mean frequency and skin-to-skin times are shown graphically in Figure 3.

**Figure 4:**
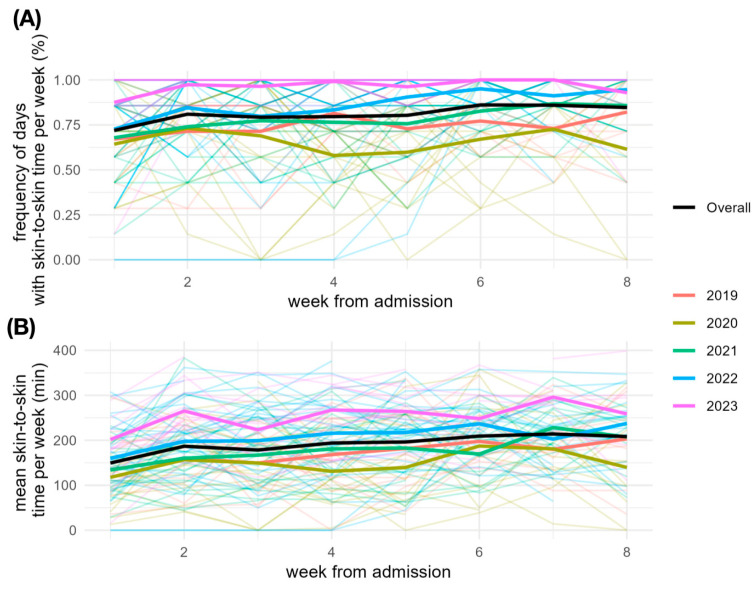
Graphical representation of the frequency of days with skin-to-skin care (**A**) and the mean skin-to-skin time per visiting day (**B**) per week in the years studied. The thin lines represent the individual preterm infants, the thick lines represent the mean value in the respective years.

Table 2 and Figure 4 show the visiting days per week and the skin-to-skin time per visiting day during the first eight weeks of the preterm infants’ lives in the years studied in tabular and graphical form.

The first electronically documented skin-to-skin contact took place on average on the 1.6th day of life (±4.94).

## 4. Discussion

The results have shown that a mean skin-to-skin time of around 4 h per visiting day was observed in Salzburg over the years. Skin-to-skin care was possible on a mean value of 8 out of 10 days. The maximum was in 2023 with skin-to-skin units on 9 out of 10 visit days with a mean skin-to-skin care time of 5 h. The rapid onset of skin-to-skin care in the first days of life is also particularly noteworthy although, as described in the methods, the first skin-to-skin contact immediately after birth was not taken into account. Already in the first week, a mean skin-to-skin time of 2.5 h per visiting day was documented, which rose to 3.5 h in the second week. Here, too, the maximum was 3.5 h per day in the first week in 2023. Despite studies showing that parental presence in neonatal intensive care units significantly improves the outcome for infants [7,14]. The WHO therefore recommends Kangaroo Care for 8–24 h a day for small premature infants [4]. There is good data on Kangaroo Care or skin-on-skin times for larger premature infants with a birth weight of >1000 g [15]. These studies also report skin-on-skin or Kangaroo Care times that come close to the WHO recommendations [14]. However, there is little data available that provides information on the exact duration of parental presence for very small preterm infants, as examined in this study [13]. A study of a neonatal intensive care unit at the Washington University School of Medicine in St. Louis found that parents were present on the unit for a mean value of 19 h per week [16]. This corresponds to approx. 2.7 h of visiting time per day. Premature infants in particular are considered especially vulnerable and are all the more dependent on parental skin-to-skin care [17]. At the same time, however, they also belong to the group with the least kangaroo care time [18]. The fear of implementing kangaroo care is still a limiting factor, especially for small and unstable infants, even though the benefits are now recognized [15,19]. The skin-to-skin times presented in this study can be classified as high overall, although they are well below the daily Kangaroo Care times recommended by the WHO [20]. This can be explained by the fact that all data in this study only include skin-to-skin times. In contrast, the times described in the literature either refer only to parental presence time in the ICU or kangaroo care times are mentioned [14]. In addition to skin-to-skin time, parental presence also includes diapering, feeding and positioning in the incubator, which the parents perform together with the nursing staff several times a day. According to the WHO definition, Kangaroo Care includes continuous and prolonged skin-to-skin contact between the mother (or another caregiver) and the baby as well as exclusive breastfeeding [4]. As smaller premature babies are not able to breastfeed independently due to their immaturity, supportive measures for exclusive breastfeeding or feeding with breast milk are also included in Kangaroo Care [20]. For this reason, the times for expressing and supplying the expressed breast milk must actually be included in the Kangaroo Care times in addition to the skin-to-skin time. In addition, medical consultations with parents take place also outside of Kangaroo Care. This shows that the parents’ actual attendance time on the ward in Salzburg is significantly longer than the skin-to-skin time. Conversely, this means that with the visiting times stated in the literature, the skin-to-skin time must be significantly lower in comparison. This is substantiated, for example, by the work of Lazarus et al. [7]. This study describes a mean skin-to-skin time of only 70 min on a mean value of less than 2 days per week, which is only a fraction of the time spent with skin-to-skin care in Salzburg. Kangaroo Care is promoted on the neonatal intensive care unit in Salzburg through the active support of the entire staff and the successful integration of the “Newborn Individualized Development Care and Assessment Program” (NIDCAP), which emphasizes the importance of skin-to-skin care. The somewhat less pronounced skin-to-skin time of the infants in the first week of life at the Salzburg neonatology unit could be explained by the limited mobility of the mothers, who often gave birth by caesarean section after a high-risk pregnancy. It can therefore be assumed that they are less mobile in the first few days after birth and still need medical care themselves. However, the rapid increase in skin-to-skin time from the second week onwards illustrates the rapid and successful implementation of Kangaroo Care in the first few days of life.

The results show that the frequency of visit days decreased significantly in the first year of the pandemic in 2020. While premature babies received skin-to-skin care on an average of ¾ of the days before the pandemic in 2019, this proportion fell to ⅔ in 2020. However, this decline did not prove to be statistically significant. From 2022, there was a recovery in visit days. In 2023, parents and premature infants had skin-to-skin contact on 9 out of 10 days. The differences between the years 2020 (in COVID-19) and 2023 (after COVID-19) were statistically significant. In contrast, the average skin-to-skin time per visit day remained largely constant over the COVID-19 years but then increased significantly. According to the literature, the COVID-19 pandemic was accompanied by strict restrictions on visiting times both inside and outside the hospital [12]. A significant decline in parental presence in neonatal intensive care units was observed worldwide [12]. Neonatal intensive care units that were unable to offer separate rooms for parents to stay in were particularly affected. Furthermore, a reduced range of supportive care services led to fewer direct interactions between parents and preterm infant [21]. The results of the study conducted here also reflect the effects of the strict pandemic-related restrictions. Quarantine requirements and more difficult access to the clinic were factors that negatively influenced the frequency of visiting days, but not the duration of the skin-to-skin period itself. It can be seen that as long as parents visited their preterm infants, extensive skin-to-skin times could still be granted.

The recovery in the frequency of visiting days from 2022 could be due to the easing of COVID-19 measures and the opening of the Ronald McDonald House in 2022. As a result of the study, a significant increase in both the frequency of visits (*p* < 0.001) and the duration of skin-to-skin time (*p* < 0.001) was found after the Ronald McDonald House opened. While parents had skin-to-skin contact with their preterm infants for an average of 3.5 h per visiting day before the Ronald McDonald House was built, this time increased to an average of 4.5 h per visiting day after the house was built. The mean frequency of days with skin-to-skin time before 2022 was 71%. After the opening, the mean frequency increased to 88%. The percentage of days spent with skin-to-skin care thus increased by around 24% after the Ronald McDonald House was built. The literature describes that a large physical distance between home and hospital is one of the most common obstacles to parental visiting time in neonatology [18]. The provision of accommodation is therefore considered to be of great importance [10]. Ronald McDonald houses help parents to live in close proximity to the intensive care unit. However, there are still no studies that quantify exact changes in visiting times in connection with Ronald McDonald Houses. However, it is described that the subjective satisfaction regarding proximity to the infant increases according to surveys [11]. The results suggest that physical proximity to the preterm infant not only allows for more frequent visits but also motivates people to stay with the infant for longer. The opening of the Ronald McDonald House in 2022 significantly improved parental accommodation at Salzburg University Hospital. Whereas previously there were only small and uncomfortable rooms for parents in the intensive care unit, the new building offers more space and better conditions for parents to stay with their infants. The initial subjective impression that skin-to-skin times would even decrease after the opening of the Ronald McDonald House was ultimately not confirmed in the objective analysis. Although the previous accommodation of parents was limited and often inadequate, their direct location on the intensive care unit possibly gave the impression of a higher presence. As parents were not only present as visitors in the NICU, but also lived there, they were also seen when they were not with their infant. This could have reinforced this impression. A novelty of the Ronald McDonald House in Salzburg should also be emphasized. Normally, these homes are reserved for parents whose place of residence is “far” away from the hospital. As a rule, a distance of at least 40 km is required. In Salzburg, parents whose place of residence is much closer to the hospital can also be accommodated. There is therefore no restriction regarding the distance to the place of residence. It can be assumed that mothers will only come to Kangaroo Care at night if they are in the immediate vicinity of their infant, as even a short journey is often a decisive obstacle. The Ronald McDonald House in Salzburg is directly connected to the neonatology department by a short, covered walkway. The fact that this concept works is impressively demonstrated by the data collected.

The mean skin-to-skin time per visit day for Primipara was 4.3 h. This means that the difference to Multipara with a mean value of 3.6 h does not appear to be particularly large. Nevertheless, the test showed a statistically significant difference (*p* = 0.043). Consequently, primipara spend a mean value of 40 min more time with skin-to-skin care per visiting day than multipara. If the frequency of visiting days during the inpatient stay is considered, it can be seen that parents with children at home visit their preterm infant in hospital just as often as parents without children at home with 80% vs. 78%. This difference was not statistically significant. The literature states that parental presence in some hospitals is significantly reduced by caring for siblings at home [13]. The present results only support this to a limited extent. It could be shown that parity has no significant influence on the frequency of visits. In the neonatal intensive care unit at the University Hospital Salzburg, parents are given maximum flexibility as to when and how often they visit their infants. Without restricted time slots, visits can be individually adapted to family and home commitments. This does not affect the frequency of possible visiting days. However, the decrease in the mean skin-to-skin time per visiting day for multipara is probably due to the fact that the additional commitments at home only allow for a shorter skin-to-skin duration. Parents who do not have to care for a child at home have more leeway to stay longer in the clinic. In the population studied, the average birth weight was 754 g and the gestational age was 25 weeks. These parameters remained constant over the years observed, as did the mean length of stay of 67 days in the intensive care unit. Consequently, the selected patient group is comparable in terms of the care required over the period from 2019 to 2023. The demographic data of our population also make it clear that our analysis is dedicated exclusively to the most vulnerable patient group in a neonatal intensive care unit.

### Limitations

The deliberate decision to take a closer look at a particularly vulnerable patient group inevitably led to a limited sample size. With a minimum of 10 premature babies in 2019 and a maximum of 24 premature babies in 2022, the statistical significance is therefore reduced. The generalizability of the results must therefore be considered limited to a certain extent. Furthermore, in addition to the fundamental limitations of a retrospective data analysis, a change in the documentation method in 2022 could have influenced the results. The skin-to-skin times were recorded in a different place in the documentation from this point onwards. As a result, the skin-to-skin times before COVID-19 may have been slightly underestimated. However, this limitation does not apply to the period from the beginning of the pandemic, as the documentation in this section remained unchanged. A third limitation is that, as described in the methods, the first skin-to-skin episode immediately after birth was not recorded electronically and therefore could not be included in this study. As a result, the onset of the first skin-to-skin contact is overestimated and the mean skin-to-skin time in the first week of life is underestimated. However, this should only be of minor importance for the interpretation of the data.

## 5. Conclusions

The study examined extremely preterm infants, who are the most vulnerable in neonatology. Existing studies show data and benefits of skin-to-skin care in less premature infants. In contrast, there is little data for extremely immature preterm infants. Due to reservations, there is a lack of randomized controlled trials that include extremely preterm infants. However, the present study shows that skin-to-skin care can be implemented from day one even in extremely preterm infants and that skin-to-skin times close to WHO recommendations are possible even in the most immature preterm infants.

The study underlines the importance of placing parents close to the neonatal intensive care unit. It shows that development-promoting measures are most effective when the infrastructural conditions are optimal. Therefore, when planning and developing care facilities for premature babies, care should be taken to ensure that short distances to the preterm infant are guaranteed at all times. Access to the Ronald McDonald House should not be reserved for parents from far away places, as even short distances can restrict visiting times. The flexibility offered to parents in the neonatal unit in Salzburg allowed parents with multiple children to visit the ICU as frequently as parents without other commitments. Although the frequency of skin-to-skin care decreased during the COVID-19 pandemic, skin-to-skin time remained constant when parents were able to visit the clinic. During a pandemic, maintaining the bond between parents and their preterm infant is crucial, and contingency plans should ensure uninterrupted parental closeness rather than initially separating parents from their preterm infants.

## Figures and Tables

**Table 1:** Mean value (Standard deviation) of frequency of days with skin-to-skin care (%) and mean skin-to-skin time per day (min). “Overall” was not considered in the *p*-values.

Year	2019	2020	2021	2022	2023	*p*-Value	Overall
frequency of days with skin-to-skin time per week (Mean (SD))	74.6% (±9.7)	64.1% (±15.1)	76.5% (±10.2)	85.8% (±16.6)	91.6% (±14.6)	<0.001	79% (±16.8)
mean skin to-to-skin time per visiting day (Mean (SD))	231 min (±56)	211 min (±64)	213 min (±55)	248 min (±62)	307 min (±124)	0.007	241 min (±83)

**Table 2:** Mean value and Standard deviation of frequency of days with skin-to-skin time (%) and mean Skin-to-skin time per day (min) during the first eight weeks of life.

Week	Frequency of Days with Skin-to-Skin Time per Week (%)		Skin-to-Skin Time per Visiting Day (Min)		n
	Mean	SD	Mean	SD	
1	71.9	26.3	152	83	88
2	81	24	204	113	84
3	79.3	28.2	200	118	84
4	79.6	25.1	203	113	84
5	80.3	23.9	215	123	82
6	86.1	18.9	231	119	73
7	85.9	18.7	235	116	66
8	84.7	24.6	237	136	54

## Data Availability

All data on which the study is based are available in anonymized form via the corresponding author.
